# Prognostic Nomograms for Nonelderly Adults with Gastric Signet Ring Cell Carcinoma

**DOI:** 10.1155/2021/1274527

**Published:** 2021-03-24

**Authors:** Hui Wang, Yao Peng, Qi Huang, Jingjing Wu, Mingjun Zhang

**Affiliations:** ^1^Anhui Medical University, Hefei, Anhui, China; ^2^Department of Oncology, The Second Affiliated Hospital of Anhui Medical University, Hefei, Anhui, China

## Abstract

**Background:**

Nomograms were established to predict the survival for gastric signet ring cell carcinoma (GSRC) in young and middle-aged adults. *Material and Methods*. Eligible patients with GSRC from 2004 to 2015 were collected from the Surveillance, Epidemiology, and End Results (SEER) database and then divided into a training and a testing cohort in proportion. Independent prognostic factors were picked by univariate and multivariate Cox regression analysis to set up nomograms. The predictive effect and clinical value of nomograms were evaluated by the concordance index (C-index), calibration curves, and receiver operating characteristic curve (ROC).

**Results:**

A total of 1686 GSRC patients were subsumed into this case for analysis, including a training (*n* = 1180) and a testing cohort (*n* = 506). Independent risk factors related to overall survival (OS) and cancer-specific survival (CSS) comprised of race, TNM stage, tumor size, number of positive lymph nodes (PLNE), and chemotherapy. For OS, the C-indexes of the training and testing cohorts were 0.737 and 0.752, while for CSS, C-indexes were, respectively, 0.749 and 0.751. These revealed that nomograms accurately predicted OS and CSS. Calibration curves and ROC demonstrated the apparent superiority of nomograms.

**Conclusion:**

We built a well-understood and comprehensive prognostic assessment model for GSRC, which provided an individualized survival prediction in the form of a quantitative score that can be considered for clinical practice.

## 1. Introduction

Gastric carcinoma (GA) is part of the most common malignant tumors of the alimentary canal with high morbidity and mortality. According to the statistics, there were approximately 1.033 million new cases and 783,000 deaths worldwide in 2018, seriously endangering human health [1]. Gastric signet ring cell carcinoma (GSRC) is a unique histopathological type of GA, which is rich in distinctive intracytoplasmic mucin components and compression of the surrounding nucleus [2]. The incidence of GSRC increased about tenfold in the United States from 1970 to 2000 (1973: 0.1/100,000; 2000: 1.4/100,000), with an average annual increase of 6.5 percent [3].

Relative to non-GSRC, GSRC is characterized by substantial invasion, rapid progression, extreme risk of metastasis, and worse prognosis [4]. Young and middle-aged (nonelderly adults) patients are the predominant group of GSRC [5]. The younger GC patients manifest more aggressive behavioral features than older, just like lower curability rate, more inadequate histologic differentiation, and challenging to make a correct diagnosis in the early stage [6–9]. At present, nearly none of the research has considered on the survival of GSRS in nonelderly adults. Therefore, it is necessary to analyze the prognosis of this population, to facilitate clinical individualized treatment and management.

Although the American Joint Committee on Cancer- (AJCC-) TNM staging system is the primary basis for evaluating the prognosis and remedy decision of cancer patients, it still has some shortcomings. For example, the effects of additional tumor factors have not been taken into account [10]. Nomogram, as a practical application tool, makes an individual prediction by quantifying risk factors into scores and shows more accurate and useful than the AJCC-TNM staging system in a variety of cancers [11–13].

Surveillance, Epidemiology, and End Results (SEER) database gathers data on morbidity, diagnosis, treatment, and survival in nearly 27.8 percent of the U.S. population. It is a relatively authoritative platform for studying the general peculiarity of carcinoma [14]. Based on the SEER, this study explored the factors influencing prognosis in 18-64 years old patients with GSRC, then attempted to develop nomograms for predicting cancer-specific survival (CSS) and overall survival (OS) of such patients, hoping to assist medical professionals to make more personalized and correct judgment in clinical practice.

## 2. Patients and Methods

### 2.1. Patients

We selected suitable patients from the SEER database (1975-2016 varying) using the SEER∗Stat software (version 8.3.6, http://seer.cancer.gov/seerstat/).

The specific inclusion criteria were (1) the period of diagnosis was from 2004 to 2015; (2) the histological pathologic confirmation was limited to adenocarcinoma, which was further stratified as signet ring cell carcinoma (SRC) (using ICD-O-3 histology code, 8490/3) or non-SRC of the adenocarcinoma (8140/3); (3) the primary site was limited to stomach (C16.0–16.9); and (4) the age ≥ 18 years old and <65 years old.

The exclusion criteria were (1) more than one primary tumor or combined with other tumors, (2) patient survived less than one month or was unknown, and (3) the necessary information covered (as follows) was lacking.

### 2.2. Clinical Variables

For each case, tumor stage was redescribed according to the 7th AJCC-TNM staging system.

The following clinicopathological variables were extracted: race, sex, pathologic grade, marital status, age at diagnosis, year of diagnosis, primary site, TNM stage, tumor size, number of positive lymph nodes (PLNE), radiotherapy (RT), chemotherapy, cause-specific death classification, survival months, survival status, and so on.

Race categories were split into black, other (including Chinese, Japanese, Korean, and Native Hawaiian/Pacific Islander and those who reported multiple categories), and white. The primary site was further subdivided into the proximal third (C16.0 cardia and C16.1 fundus), the mid third (C16.2 body and C16.5 lesser curvature), the distal third (C16.3 antrum and C16.4 pylorus), greater curvature (C16.6), overlapping lesions of the stomach (C16.8), and NOS (C16.9).

### 2.3. Survival Analysis

The survival curves of OS and CSS were drawn by Kaplan-Meier function, and log-rank was used to test the difference in survival rate among different groups.

### 2.4. Construction of the Nomograms

All applicable patients were randomized into the training and testing cohort in a ratio of 7 : 3. Nomograms were designed by the training cohort. Univariate and multivariate Cox regression analyses were used to screen out and determine significant independent prognostic risk factors for CSS and OS. Then, we incorporated these factors into construction nomograms.

### 2.5. Validation of the Nomograms

Validation of the nomograms was primarily implemented in both the training and testing cohorts. The Harrell's concordance index (C-index) and calibration curves were applied to evaluate the predictive performance of the nomograms. The higher the Cindex was, the more accurate the prediction. Calibration curves were generated to visually judge the consistency between the predicted and actual probability of survival on the basis of bootstrap 1000 resamples [15–17]. The receiver operating characteristic curve (ROC) and the area under ROC (AUC) were also used to assess the precision and specificity of nomograms.

Survival rates were calculated using SPSS 22.0 Statistical Package, the age-adjusted incidence of GSRC aged 15-64 years from 2004 to 2015 was analyzed by GraphPad Prism 8, and the rest part of statistical analysis was undertaken using the R software (version 3.6.1, http://www.r-project.org/). The *P* value <0.05 was deemed statistically significant.

## 3. Results

### 3.1. Patient Characteristics

1686 suitable cases with GSRC and 3060 instances with non-GSRC were contained in this process from 2004 to 2015 (Supplementary Table 1).  Table 1 presented basic information about GSRC in both training (*n* = 1180) and testing (*n* = 506) cohorts, including demographics, clinical characteristics, and treatment condition. In terms of the entire study cohort, there were 879 males and 807 females. White (64.8%), married (66.4%), middle-aged (23.8%), and poor differentiation (93.7%) accounted for predominance. With regard to the primary site of tumor, the most familiar location was the distal third gastric region, occupying approximately 33.3%, followed by the mid third gastric region. In addition to chemotherapy, which became even more common therapy for all patients, and many patients had also received RT.

**Figure 1:**
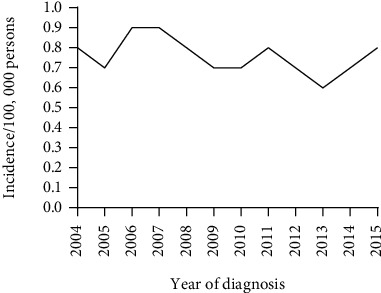
Annual age-adjusted incidence of gastric signet ring cell carcinoma from 2004 to 2015 in 15-64 years old.

Figure 1 revealed the age-adjusted incidence of GSRC in 15-64 years old from 2004 to 2015, showing a relatively stable rate of 0.6-0.9/100,000 persons. We can more intuitively understand the incidence trend of the disease in nonelderly patients.

### 3.2. Survival

**Figure 2:**
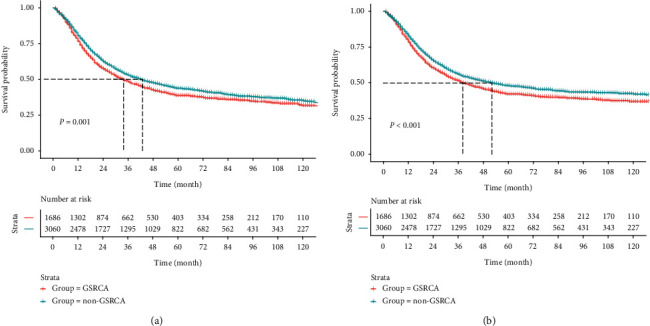
Survival analysis of GSRC and non-GSRC: (a) OS and (b) CSS were shown for all patients.

In the whole cohort, at the end of follow-up, 993 patients died in all. Kaplan-Meier survival analysis displayed that the OS rates of patients with GSRC in 3, 5, and 10 years were 48.5%, 38.7%, and 31.6%, and the CSS rates were 51.2%, 42.1%, and 36.7%, respectively. Median OS and CSS were 34 and 38 months. The OS rates of 3, 5, and 10 years were 52.9%, 43.6%, and 35.1% who were non-GSRC patients, and the CSS rates were 55.7%, 47.7%, and 42.1%. GSRC acquired a more unfavourable outcome than non-GSRC in 18-64 years old (Figure 2(a), OS; Figure 2(b), CSS).

**Figure 3:**
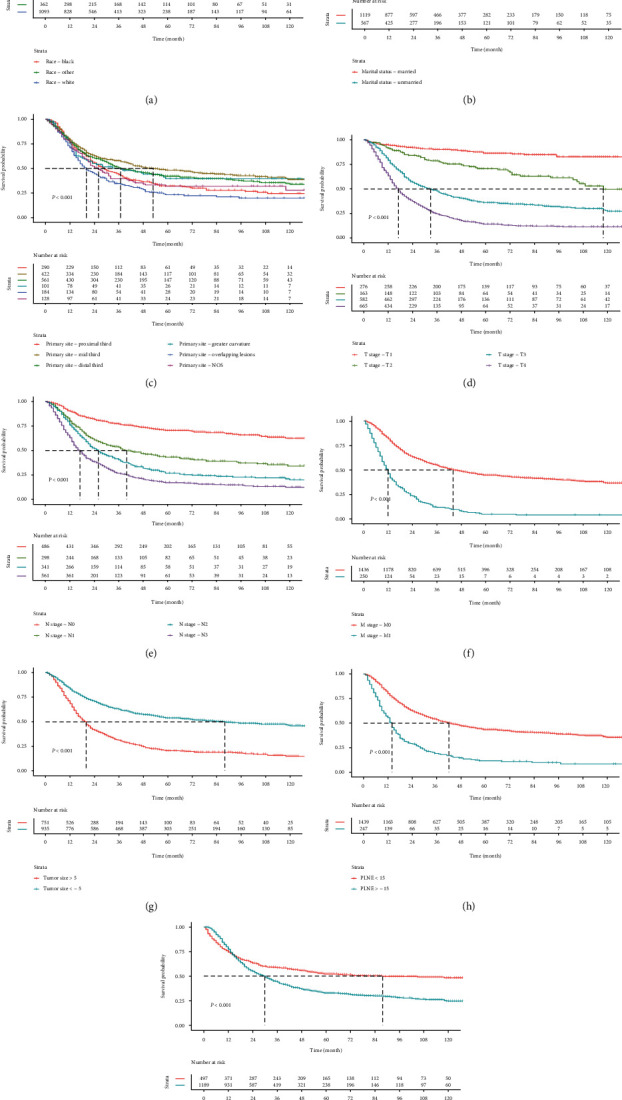
Kaplan-Meier survival analysis of OS for GSRC stratified by (a) race, (b) marital status, (c) primary site, (d) T stage, (e) N stage, (f) M stage, (g) tumor size, (h) PLNE, and (i) chemotherapy.

**Figure 4:**
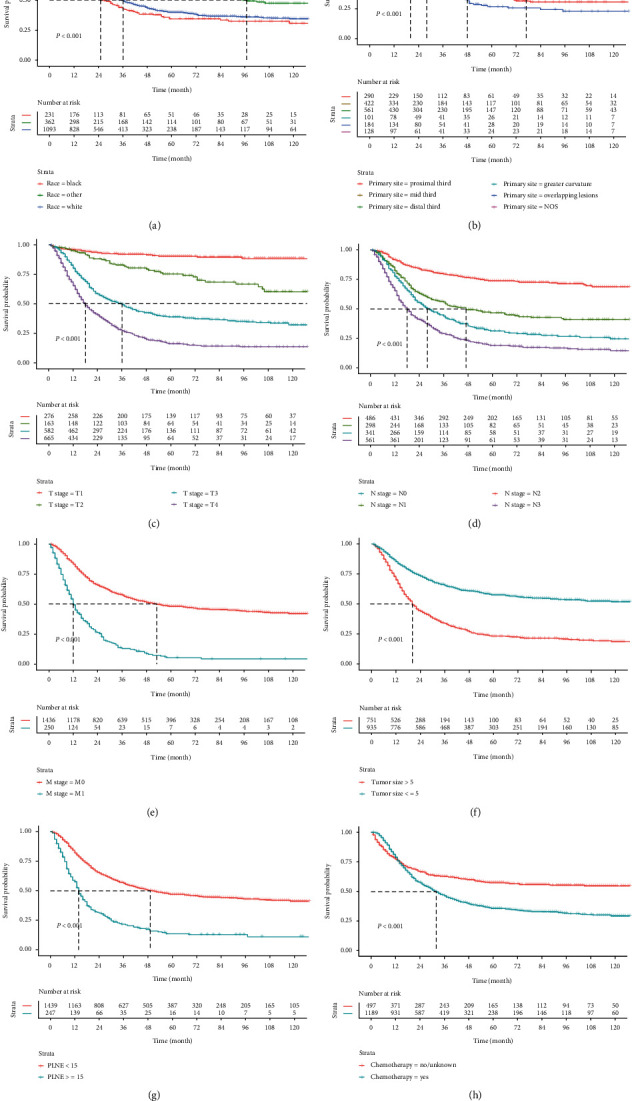
Kaplan-Meier survival analysis of CSS for GSRC stratified by (a) race, (b) primary site, (c) T stage, (d) N stage, (e) M stage, (f) tumor size, (g) PLNE, and (h) chemotherapy.

Figures 3 and 4 severally discovered the Kaplan-Meier survival curves of OS and CSS for some pathological factors of GSRC, which were meaningful to survival. Patients whose primary site was located in the mid third gastric region, the OS and CSS were longer. When the tumor was progressed, the PLNE was increased, and the prognosis was poorer. Race and chemotherapy were also important factors affecting survival. Married patients inclined to gain better OS than unmarried patients but did not correlate with CSS. Besides, sex, grade, RT, and age at diagnosis were no significant impact on OS or CSS.

### 3.3. Construction of Prognostic Nomograms for OS and CSS

Through the univariate and multivariate Cox regression analysis, finally, we obtained seven independent prognostic risk factors of OS and CSS, including race, TNM stage, tumor size, PLNE, and chemotherapy (Table 2, OS; Table 3, CSS).

**Figure 5:**
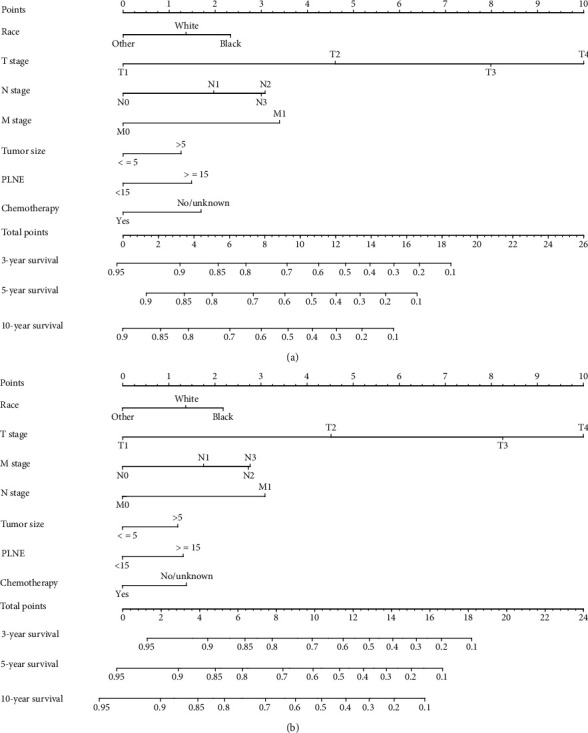
Nomograms for predicting the 3-, 5-, and 10-year OS (a) and CSS (b) of patients with GSRC in 18-64 years old.

In the whole study set, nomograms were established according to all of the independent prognostic factors stemmed from multivariate Cox regression analysis in the training cohort. We were able to intuitively estimate the probabilities of OS (Figure 5(a)) and CSS (Figure 5(b)) for 3, 5, and 10 years by adding the scores related to each variable and predicting the total points to the bottom.

### 3.4. Validation of Prognostic Nomograms for OS and CSS

The nomograms which were validated showed excellent accuracy. When validation of nomograms was performed in the training cohort for OS and CSS, the C-indexes were, respectively, 0.737 and 0.749. The C-indexes for the nomograms to predict OS and CSS were, respectively, 0.752 and 0.751 in the testing cohort.

**Figure 6:**
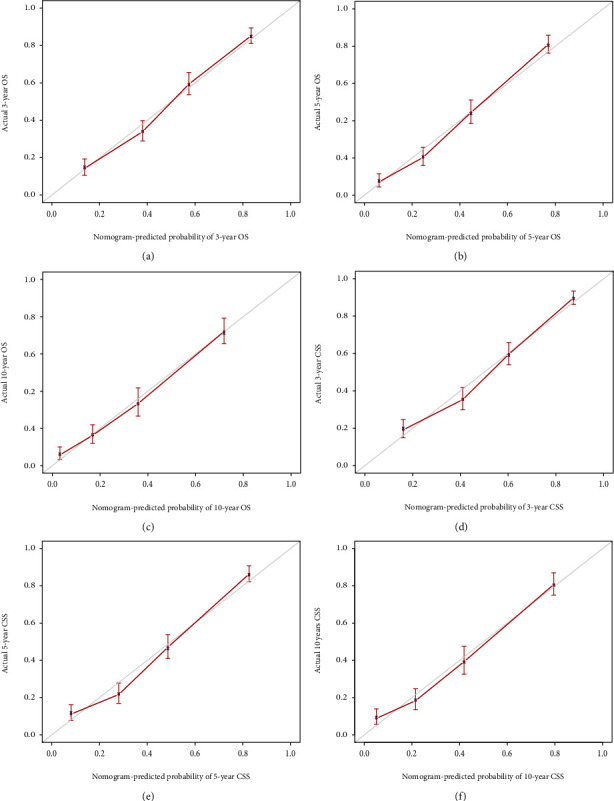
Calibration curves of the nomogram for 3-, 5-, and 10-year OS (a–c) and CSS (d–f) in training cohort.

**Figure 7:**
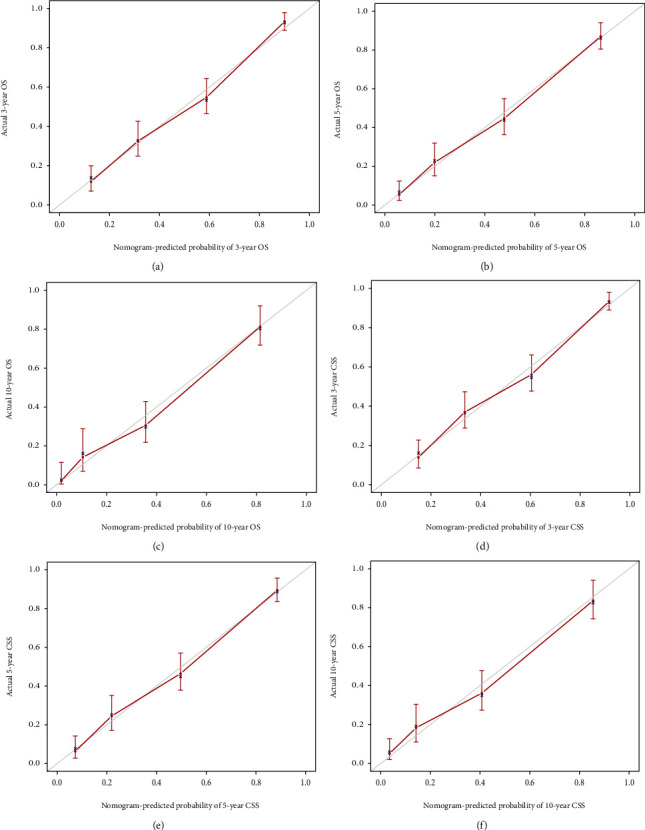
Calibration curves of the nomogram for 3-, 5-, and 10-year OS (a–c) and CSS (d–f) in testing cohort.

**Figure 8:**
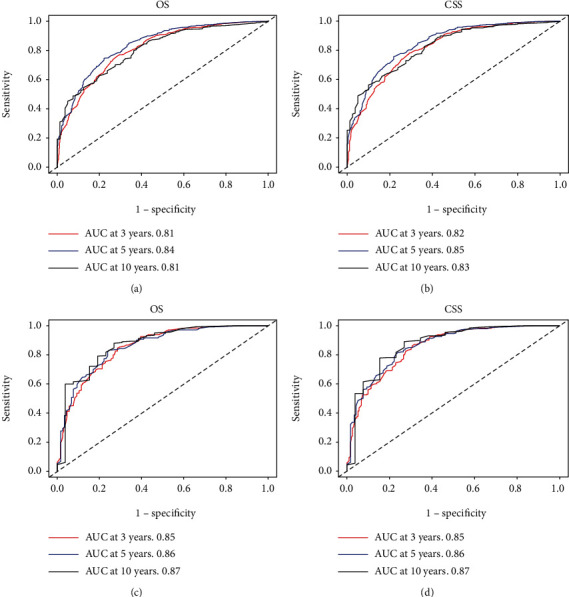
ROC curves for 3-, 5-, and 10-year OS and CSS in training (a, b) and testing (c, d) cohort.

Through the calibration curves for probabilities of OS and CSS, predictions by the nomograms were in optimal correlation with actual survival observation in training (Figure 6) and testing cohort (Figure 7).  Figure 8 illustrated that the values of AUC indicated a satisfactory ability to predict survival, which was all over 0.8 (0.81-0.85 in the training cohort; 0.85-0.87 in the testing cohort). To sum up, nomograms that we built showed considerable reliability.

## 4. Discussion

GSRC is a highly malignant type of GA, with a reported five-year survival rate of only 15.9% [18]. The age of high incidence is generally less than 65 years old in GSRC. Nevertheless, survival analysis of this population continues to be scarce. Hence, it is urgent to make an in-depth study on the prognostic factors and establish a predictive model to guide the clinical work better. Thus, on the strength of a large population cohort, we described the prognostic factors and constructed nomograms to predict OS and CSS for GSRC patients aged 18-64 years.

For the Asians, Native Americans, and Pacific Islanders, the prognosis was slightly better than blacks and whites. This was in line with the research by Wang et al. [19]. Several studies had come to similar conclusions. Kim et al. studied 13084 patients with gastric adenocarcinoma in the Los Angeles County Cancer Surveillance Program and found that the prognosis had improved in Asian patients. All of these patients received surgery in this county, basically except for the survival effects of radical gastrectomy and expanded lymphadenectomy, which were more common in Asia [20]. Beyond all doubt, TNM stage and PLNE are internationally recognized prognostic factor.

Tumor size reflects tumor burden of tumor patients and is related to the burden and prognosis. A study of 946 patients with GSRC showed that the OS of small tumors (diameter less than 49 mm) was better than that of large tumors (diameter greater than 49 mm), and tumor size was an independent prognostic factor. In addition, lymph node metastasis, distant metastasis, and poor differentiation are the most likely to occur in the larger volume of GSRC, and these factors will affect the survival of patients [8, 21, 22].

In recent years, the psychological intervention has gradually received attention in cancer treatment. Similar to our research results, many literatures pointed out that marital status was a socioeconomic factor that affects prognosis of cancer patients [23–26]. This may be married people get more humanistic care and emotional support to achieve better survival outcomes [27]. Although it was not an independent risk factor, it should be taken seriously.

Needless to state, surgery is the primary treatment for patients with GA, but many studies had proposed that chemotherapy can improve the prognosis of patients. Recent data indicated that paclitaxel-based chemotherapy had a definite effect in gastric signet ring cell carcinoma [28–30]. The advanced stage GSRC receiving TEFOX (docetaxel-5FU-oxaliplatin) chemotherapy had an effective rate of 65% and a median OS of up to 14 months [31]. We discovered that chemotherapy was an adverse prognostic factor by univariate COX regression; however, in multivariate COX regression, it was protective. Therefore, we analyzed the variables affecting chemotherapy and then found some significant differences between chemotherapy group and nonchemotherapy group, which were primary site, T stage, N stage, RT, and tumor size. Further stepwise Cox regression showed that the N stage notably affected chemotherapy. That was to say, among the single factors, the worse prognosis of chemotherapy patients may be due to their later N stage. Consistent with our study, some articles also put forward that N stage was more senior in chemotherapy patients [32–35]. Despite the fact that GSRC is relatively insensitive to non-GSRC chemotherapy, it may benefit from specific chemotherapy regimens.

But for all this, our research still has several limitations that need to be pondered. First of all, in the SEER database, some important biochemical indexes were not explained, such as carcinoembryonic antigen (CEA) and carbohydrate antigen 19-9 (CA19-9). Differences in these factors may lead to differences in survival. Secondly, the information related to chemotherapy was not fully recorded, including chemotherapy regimen used, drugs dose, treatment duration, and adverse reaction. So it is not possible to find the best personal treatment. Additionally, we excluded some patients who were short of some information, which may lead to a deviation in prognosis analysis. Anyway, our nomograms were reliable individualized prediction models.

## 5. Conclusion

In conclusion, we firstly established and validated the nomograms for nonelderly adults with primary GSRC, which were used to forecast OS and CSS. Furthermore, the nomograms revealed excellent performance and strong predictive ability. This will provide theoretical support for the formulation of clinical treatment programs and prognostic judgment.

## Figures and Tables

**Table 1:** Patient demographics and clinical characteristics of gastric signet ring cell carcinoma (GSRC) in training and testing cohort.

Variables	All patients	Training cohort	Testing cohort
Number of patients, *n* (%)	1686 (100)	1180 (70)	506 (30)
Age at diagnose (years)			
18-44	401 (23.8)	273 (23.1)	128 (25.3)
45-64	1285 (76.2)	907 (76.9)	378 (74.7)
Race			
Black	231 (13.7)	156 (13.2)	75 (14.8)
Other	362 (21.5)	253 (21.5)	109 (21.6)
White	1093 (64.8)	771 (65.3)	322 (63.6)
Sex			
Female	807 (47.9)	573 (48.6)	234 (46.2)
Male	879 (52.1)	607 (51.4)	272 (53.8)
Marital status			
Married	1119 (66.4)	785 (66.5)	334 (66.0)
Unmarried	567 (33.6)	395 (33.5)	172 (34.0)
Primary site			
Proximal third (cardia and fundus)	290 (17.2)	206 (17.4)	84 (16.6)
Mid third (body and lesser curvature)	422 (25.0)	303 (25.7)	119 (23.5)
Distal third (antrum and pylorus)	561 (33.3)	383 (32.5)	178 (35.2)
Greater curvature	101 (6.0)	73 (6.2)	28 (5.5)
Overlapping lesions	184 (10.9)	130 (11.0)	54 (10.7)
NOS	128 (7.6)	85 (7.2)	43 (8.5)
Grade			
Moderately differentiated (II)	37 (2.2)	25 (2.1)	12 (2.4)
Poorly differentiated (III)	1579 (93.7)	1104 (93.6)	475 (93.9)
Undifferentiated (IV)	70 (4.1)	51 (4.3)	19 (3.7)
T stage			
T1	276 (16.4)	182 (15.5)	94 (18.6)
T2	163 (9.7)	109 (9.2)	54 (10.7)
T3	582 (34.5)	426 (36.1)	156 (30.8)
T4	665 (39.4)	463 (39.2)	202 (39.9)
N stage			
N0	486 (28.8)	322 (27.3)	164 (32.4)
N1	298 (17.7)	213 (18.1)	85 (16.8)
N2	341 (20.2)	248 (21.0)	93 (18.4)
N3	561 (33.3)	397 (33.6)	164 (32.4)
M stage			
M0	1436 (85.2)	1005 (85.2)	431 (85.2)
M1	250 (14.8)	175 (14.8)	75 (14.8)
Tumor size (cm)			
≤5	935 (55.5)	651 (55.2)	284 (56.1)
>5	751 (44.5)	529 (44.8)	222 (43.9)
PLNE			
<15	1439 (85.3)	1001 (84.8)	438 (86.6)
≥15	247 (14.7)	179 (15.2)	68 (13.4)
Radiation			
Yes	725 (43.0)	513 (43.5)	212 (41.9)
No/unknown	961 (57.0)	667 (56.5)	294 (58.1)
Chemotherapy			
Yes	1189 (70.5)	837 (70.9)	352 (69.6)
No/unknown	497 (29.5)	343 (29.1)	154 (30.4)

**Table 2:** Univariate and multivariate Cox analysis of the determinants of overall survival (OS) of patients with gastric signet ring cell carcinoma (GSRC).

Variables	Univariate analysis		Multivariate analysis	
	HR (95% CI)	*P* value	HR (95% CI)	*P* value
Age at diagnose (years)				
18-44	1			
45-64	1.118 (0.937-1.334)	0.215		
Race				
Black	1		1	
Other	0.672 (0.517-0.873)	0.003	0.658 (0.501-0.864)	0.003
White	0.948 (0.765-1.174)	0.624	0.870 (0.694-1.090)	0.226
Sex				
Female	1			
Male	0.974 (0.840-1.129)	0.722		
Marital status				
Married	1		1	
Unmarried	1.210 (1.036-1.412)	0.016	1.108 (0.943-1.301)	0.214
Primary site				
Proximal third (cardia and fundus)	1		1	
Mid third (body and lesser curvature)	0.741 (0.588-0.934)	0.011	0.815 (0.642-1.035)	0.094
Distal third (antrum and pylorus)	0.882 (0.713-1.092)	0.249	0.831 (0.664-1.040)	0.106
Greater curvature	0.932 (0.661-1.313)	0.687	0.887 (0.625-1.258)	0.500
Overlapping lesions	1.314 (1.012-1.705)	0.040	0.808 (0.613-1.065)	0.130
NOS	0.985 (0.712-1.362)	0.927	0.870 (0.623-1.215)	0.413
Grade				
Moderately differentiated (II)	1			
Poorly differentiated (III)	1.230 (0.695-2.178)	0.477		
Undifferentiated (IV)	1.362 (0.701-2.645)	0.362		
T stage				
T1	1		1	
T2	2.624 (1.598-4.310)	<0.001	2.562 (1.534-4.278)	<0.001
T3	5.877 (3.951-8.740)	<0.001	4.668 (3.028-7.195)	<0.001
T4	11.257 (7.604-16.670)	<0.001	7.353 (4.740-11.406)	<0.001
N stage				
N0	1		1	
N1	2.189 (1.678-2.854)	<0.001	1.327 (1.001-1.759)	0.049
N2	2.689 (2.091-3.458)	<0.001	1.528 (1.166-2.002)	0.002
N3	4.496 (3.580-5.647)	<0.001	1.724 (1.312-2.265)	<0.001
M stage				
M0	1		1	
M1	3.547 (2.958-4.254)	<0.001	2.113 (1.746-2.557)	<0.001
Tumor size (cm)				
≤5	1		1	
>5	2.338 (2.010-2.720)	<0.001	1.276 (1.082-1.504)	0.004
PLNE				
<15	1		1	
≥15	2.800 (2.335-3.357)	<0.001	1.418 (1.135-1.771)	0.002
Radiation				
No/unknown	1			
Yes	0.909 (0.784-1.055)	0.211		
Chemotherapy				
No/unknown	1		1	
Yes	1.399 (1.176-1.666)	<0.001	0.669 (0.555-0.807)	<0.001
Year of diagnose	0.964 (0.942-0.987)	0.002	1.000 (0.976-1.025)	0.987

HR: hazard ratio; CI: confidence interval; PLNE: number of positive lymph nodes.

**Table 3:** Univariate and multivariate Cox analysis of the determinants of cancer-specific survival (CSS) of patients with gastric signet ring cell carcinoma (GSRC).

Variables	Univariate analysis		Multivariate analysis	
	HR (95% CI)	*P* value	HR (95% CI)	*P* value
Age at diagnose (years)				
18-44	1			
45-64	1.127 (0.935-1.358)	0.209		
Race				
Black	1		1	
Other	0.644 (0.487-0.853)	0.002	0.613 (0.462-0.813)	<0.001
White	0.967 (0.771-1.212)	0.769	0.859 (0.681-1.082)	0.197
Sex				
Female	1			
Male	0.969 (0.829-1.132)	0.688		
Marital status				
Married	1			
Unmarried	1.170 (0.993-1.378)	0.060		
Primary site				
Proximal third (cardia and fundus)	1		1	
Mid third (body and lesser curvature)	0.732 (0.573-0.934)	0.012	0.811 (0.630-1.045)	0.105
Distal third (antrum and pylorus)	0.880 (0.703-1.101)	0.263	0.835 (0.659-1.057)	0.134
Greater curvature	0.886 (0.613-1.279)	0.518	0.840 (0.578-1.222)	0.362
Overlapping lesions	1.347 (1.027-1.769)	0.032	0.818 (0.613-1.092)	0.174
NOS	0.961 (0.681-1.356)	0.822	0.853 (0.598-1.217)	0.381
Grade				
Moderately differentiated (II)	1			
Poorly differentiated (III)	1.207 (0.665-2.193)	0.536		
Undifferentiated (IV)	1.398 (0.701-2.791)	0.342		
T stage				
T1	1		1	
T2	2.957 (1.636-5.347)	<0.001	2.760 (1.502-5.072)	0.001
T3	7.929 (4.907-12.812)	<0.001	5.951 (3.557-9.955)	<0.001
T4	15.347 (9.540-24.688)	<0.001	9.408 (5.590-15.836)	<0.001
N stage				
N0	1		1	
N1	2.350 (1.760-3.138)	<0.001	1.345 (0.992-1.823)	0.057
N2	2.926 (2.225-3.848)	<0.001	1.555 (1.163-2.080)	0.003
N3	5.091 (3.972-6.525)	<0.001	1.780 (1.332-2.379)	<0.001
M stage				
M0	1		1	
M1	3.764 (3.119-4.542)	<0.001	2.193 (1.801-2.670)	<0.001
Tumor size (cm)				
≤5	1		1	
>5	2.448 (2.086-2.873)	<0.001	1.278 (1.076-1.519)	0.005
PLNE				
<15	1		1	
≥15	2.910 (2.410-3.515)	<0.001	1.428 (1.135-1.797)	0.002
Radiation				
No/unknown	1			
Yes	0.898 (0.768-1.051)	0.179		
Chemotherapy				
No/unknown	1		1	
Yes	1.492 (1.237-1.800)	<0.001	0.698 (0.572-0.851)	<0.001
Year of diagnose	0.958 (0.935-0.982)	<0.001	0.996 (0.971-1.022)	0.742

HR: hazard ratio; CI: confidence interval; PLNE: number of positive lymph nodes.

## Data Availability

Data extracted from the SEER database do not require individual informed consent. The patient data in this study was anonymously managed in all stages, including stages of data cleaning and statistical analyses. This study was conducted in accordance with the Declaration of Helsinki.

## Supplementary Materials

Supplementary Materials Supplementary Table 1 shows the demographics and clinical characteristics of 1686 gastric signet ring cell carcinoma (GSRC) and 3060 non-GSRC.
