# Supplementary material for: Prognostic Nomograms for Nonelderly Adults with Gastric Signet Ring Cell Carcinoma
Source: Biomed Res Int. 2021 Mar 24;2021:1274527. doi: 10.1155/2021/1274527 (PMC8016563; doi:10.1155/2021/1274527)
Supplement: Supplementary Materials — Supplementary Table 1 shows the demographics and clinical characteristics of 1686 gastric signet ring cell carcinoma (GSRC) and 3060 non-GSRC. [file 1274527.f1.pdf]

Supplementary Table 1 Patient demographics and clinical characteristics of gastric signet ring cell carcinoma (GSRC) and non-GSRC

Variables	GSRC	non-GSRC
**Number of Patients (n)**	1686	3060
**Age at Diagnose (years)**		
18-44	401	345
45-64	1285	2715
**Race**		
Black	231	432
Other	362	500
White	1093	2128
**Sex**		
Female	807	777
Male	879	2283
**Marital Status**		
Married	1119	2068
Unmarried	567	992
**Primary Site**		
Proximal third (cardia and fundus)	290	1586
Mid third (body and lesser curvature)	422	451
Distal third (antrum and pylorus)	561	613
Greater curvature	101	106
Overlapping lesions	184	142
NOS	128	162
**Grade**		
Well and moderately differentiated (I/II)	37	1137
Poorly differentiated (III)	1579	1862
Undifferentiated (IV)	70	61
**T Stage**		
T1	276	530
T2	163	182
T3	582	1314
T4	665	1034
**N Stage**		
N0	486	988
N1	298	791
N2	341	615
N3	561	666
**M Stage**		
M0	1436	2717
M1	250	343
**Tumor Size (cm)**		
⩽5	935	362
>5	751	2698
**PLNE**		
<15	1439	2864
⩾15	247	196
**Radiation**		
Yes	725	1516
No/Unknown	961	1544
**Chemotherapy**		
Yes	1189	2131
No/Unknown	497	929

Note: TNM Stage according to the 7th American Joint Committee on Cancer (AJCC) staging system
Abbreviation: PLNE, number of positive lymph nodes
